# Educated Girls

**Published:** 1881-05

**Authors:** 


### EDUCATED GIRLS.

CULTURE of the mind, we are told, unfits women for home life. Half culture may do so; but advanced mental culture will not. It has been said that the passing of a university examination is of no earthly use to women who marry. Is it, then, of no use that a woman who is married should be able in a great measure to educate her own children; to give a right bias to their minds; to answer the innumerable "Why is this?" and "what makes so and so," of little people; to teach them to think accurately; to clear up school difficulties, etc.? to say nothing of all the great rest and refreshment to her own mind to be able to enjoy books, papers, magazines, the fine arts, and to forget for a while those minor household cares to which some would entirely consign her? Is it of no earthly use that she can strengthen herself against petty, carking cares, by forgetting herself for a little time in that delightful abstract world of fancy and speculation which lies before us in books? Then let us take the case of a woman who does not marry. What is she to do? Girls cannot know at fifteen—an age when the higher education usually begins—whether they will marry or not, and those who do not are often obliged to earn their own living. How can they do that? With a half education they cannot become teachers. Little else is open to them save domestic service. Thus they become more dependent than ever on the idea of marriage.

If, indeed, there is one thing which can render a single woman independent, it is a cultivated mind, which enables her to take a larger view of life. A girl who can take pleasure in reading the best authors, in designing graceful pictures from nature, in music, in reading of the manners and customs of foreign peoples, in reading foreign authors in the original, has resources in herself that qualify her for profitable employment, and that command the respect and friendship of cultivated people.

"WHAT makes the sea salt?" said the teacher. And young America shouted, "The codfish that are in it."

### NOW.

Forenoon, and afternoon, and night! Forenoon,
And afternoon, and night! Forenoon and—
What! The empty song repeats itself. No more
Yea, that is life. Make this forenoon sublime,
This afternoon a psalm, this night a prayer,
And Time is conquered, and thy crown is won.

—*Edward R. Sill.*

